# Factors Associated with COVID-19 Vaccine Hesitancy and Case Status among New Jersey Secondary Educational Professionals

**DOI:** 10.3390/vaccines11111667

**Published:** 2023-10-31

**Authors:** Juhi Aggarwal, Kimberly T. Nguyen, Maryanne L. Campbell, Stephanie Shiau, Derek G. Shendell

**Affiliations:** 1 NJ Safe Schools Program, Rutgers School of Public Health (SPH), Rutgers University, Piscataway, NJ 08854, USAmlf159@sph.rutgers.edu (M.L.C.); shendedg@sph.rutgers.edu (D.G.S.); 2 Department of Epidemiology and Biostatistics, Rutgers School of Public Health (SPH), Piscataway, NJ 08854, USA; stephanie.shiau@rutgers.edu; 3 Department of Environmental and Occupational Health and Justice, Rutgers School of Public Health (SPH), Piscataway, NJ 08854, USA

**Keywords:** COVID-19 vaccine, safety and health, teacher, vaccination, vaccine hesitancy

## Abstract

Background: Vaccine hesitancy remains a societal problem, including during the COVID-19 pandemic. New Jersey (NJ) Safe Schools Program provides work-based learning training to supervisory-level career–technical–vocational education teachers and administrators who have to consider varied state and local mandates concerning COVID-19 vaccination and exemptions. Methods: In early 2022, we distributed an online survey via PsychData to individuals trained between 2014 and 2022 to understand NJ teachers’ practices and concerns regarding COVID-19 vaccines. Overall, 269 completed the survey. We stratified data by vaccination status, number of doses, booster status, age, teaching experience, gender, race, county of work, and COVID-19 diagnosis status. Results: Overall, results suggested differences in COVID-19-related concerns, including access to, perceptions of, and confidence in COVID-19 vaccines and COVID-19-related practices. About 90.7% received the initial vaccine; 77.7% received the booster. About half the participants had received a positive COVID-19 diagnosis by the time of the survey; they were less likely to get the vaccine or booster if they had received the initial vaccine. Conclusions: Data suggested differences in levels of COVID-19-related concerns and confidence in, or importance of, vaccines when comparing different demographic factors and vaccination practices. The data informs efforts to understand factors affecting vaccine hesitancy among educational professionals.

## 1. Introduction

For several decades, vaccine hesitancy has been a challenge for medical professionals and public health advocates. These problems have existed even outside of the coronavirus disease 2019 (COVID-19) pandemic [1]. Vaccine hesitancy is the rejection of, or the delay in, accepting vaccine administration [2]. According to a 2020 survey, there was a high level of COVID-19 vaccine hesitancy not only in the United States (U.S.) but worldwide [3]. While it has been repeatedly proven that vaccination efforts can effectively decrease the morbidity and mortality of diseases, vaccine hesitancy continues to be a persistent problem among different populations [2]. It is particularly important to protect school educational professionals, who are arguably at an increased risk of contracting preventable diseases since they work closely with school-aged children who require parental consent prior to receiving a vaccine [4]. To protect their health and safety while working in an in-person environment, New Jersey (NJ) implemented a statewide policy for the 2021–2022 school year that required secondary educational professionals to either receive the COVID-19 vaccine or undergo regular testing if they elect to remain unvaccinated before returning to the workplace [5]. On 15 August 2022, the policy no longer required unvaccinated secondary educational professionals to undergo routine COVID-19 testing [5].

Vaccines play a crucial role in society’s response to COVID-19. The U.S. Centers for Disease Control and Prevention have stated that using approved vaccines is effective in protecting users from severe illnesses, infection, and death [6]. The FDA stated that the Pfizer-BioNTech vaccine was 90.7% effective in preventing COVID-19 in children aged 5 to 11 [7]. Researchers have reported that no matter the child’s age, COVID-19-related hospitalization rates are higher among unvaccinated youth patients than vaccinated youth patients [8,9]; this is also reflected in adults. A study reported that after one dose of the vaccine, COVID-19 hospital admissions among adults decreased by 91.0% between December 2020 and February 2021 in Scotland [10]. Despite the information available, there have been parents who are advocating against receiving the vaccine for both themselves and their children [11]. This could lead to teachers and other school educational professionals being at higher risk for contracting COVID-19 [4,12,13,14,15]. In a study conducted in China, it was found that college professors were more vaccine-hesitant than their students. Many teachers were worried about the overall safety of the vaccine [12]. A study of elementary students in Greece found that only 38.1% of teachers intended to get vaccinated and those who intended to get vaccinated believed that teachers were a high-risk group for contracting COVID-19 [16]. In the U.S., a study found the biggest factors in reducing concerns about the COVID-19 vaccine included a high belief in vaccine safety, the perception of an elevated risk of getting infected, trust in the effectiveness of the vaccine, and trust in the information sources for the vaccine [12]. School educational professionals in Germany considered students to be the greatest risk factor for getting COVID-19 in the workplace [16]. Students under the age of 16 were initially delayed in accessing the COVID-19 vaccine, as on 11 December 2020 the Pfizer-BioNTech COVID-19 vaccine was approved by the U.S. Food and Drug Administration (FDA) to be used for both school educational professionals and students over the age of 16, while in May 2021, the vaccine was available to adolescents ages 12 to 15, and was then available to those between ages 5 and 11 on 29 October 2021 [17,18]. 

Researchers have also reported correlations between different socio-economic and demographic factors and vaccine hesitancy [19] among both rural and urban adults [20]. Regardless of one’s age, it is clear how impactful vaccination efforts and educational campaigns can be when attempting to limit the burden of any disease [21,22]. Due to the severe nature of COVID-19, researchers, physicians, and public health officials must continue to encourage vaccine intake, especially among high-risk populations, including teachers and other educational professionals [4]. The present study focuses on exploring the impacts and reasons for vaccine hesitancy regarding the COVID-19 vaccine among secondary or high school educational professionals working in NJ and those trained to supervise students and other staff in career–technical–vocation education (CTE) work-based learning (WBL) (formerly called school-sponsored structured learning experiences in NJ) between 2014 and 2022. Findings from this study will provide valuable insight into how mass vaccination efforts can be improved and deemed successful by determining factors that made it difficult to get the COVID-19 vaccination and what factors are important to people when considering it. Data can also inform future guidance to schools, and district’s future policies regarding vaccines.

## 2. Materials and Methods

### 2.1. Study Population

The New Jersey Safe Schools (NJSS) program provides safety and health (S&H) training to over 1000 teachers seeking work-based learning (WBL) (formerly called school-sponsored structured learning experiences in NJ) in CTE endorsement between 2014 and 2022 [23]. These teachers were asked to complete an online survey via PsychData, where a total of 269 completed or partially completed the survey. Our sample size estimated for this study was 190 for a 95% confidence level, a 5% margin of error, an 81.4% population proportion of fully or partially vaccinated people in the U.S. [24], and a population size of 1015 teachers who were eligible for our survey. This survey was distributed online in two rounds: in March–April 2022 for teachers who had finished training between September 2014 and March 2022, and again in June–July 2022 for teachers who finished the training between March and June 2022. Results from both groups were combined for the study’s analysis. 

### 2.2. Survey Details

The survey used in this study included 28 questions with multiple parts about vaccine status, experience specifically with the COVID-19 vaccine, vaccine perceptions and opinions regarding the ease of obtaining the vaccine, factors that were important to obtaining the vaccine, factors that helped convince the participants the vaccine was safe to get, self-reported COVID-19 case information (including hospitalization status), and demographics. Other factors, such as COVID-19 vaccine education, trusted sources of information, safety practices, and concerns about contracting COVID-19, were explored in a previously published study [25]. For more information on the survey questions, please read our previously published study [25]. The full survey can be found in the Supplementary Materials file.

### 2.3. Data Analysis

After data management and initial descriptive statistics were computed for the entire State of NJ study sample, for each survey question/variable the data were stratified by the vaccination status, number of doses of vaccine, and booster status at the time of the survey; gender; age; county of work; race/ethnicity; and teaching experience (both overall and in NJ). For justification of the county separation, please reference our previously published study [25]. In this study, those who did not get vaccinated are considered vaccine-hesitant. We could not state if someone had received the full dose of the vaccine as we could not determine if those who received one dose had an incomplete vaccine (one of two required doses), a vaccine that only required one dose, or were in the middle of a two-dose required schedule. Data analyses were carried out using Microsoft Excel and SAS Analytics Software 9.4 (Cary, NC, USA). Continuous variables (age and teaching experience overall and in NJ) were assessed for normality using the Kolmogorov–Smirnov test, summarized using means and standard deviations (SD). Since, in this study, continuous variables were normal, the means of the continuous variables were compared between groups using *t*-tests or ANOVA tests. Fold F-tests to determine equality of variance were conducted for all continuous variables. For those variables with equality of variance, a pooled *t*-test was used, and for those without equality of variance, a Satterthwaite *t*-test was used. Because of the small sample sizes, categorical variables were summarized using percentages and compared between groups using Fisher’s exact test. Missing data were excluded from analyses. Calculated p-values below 0.05 were originally considered statistically significant and Bonferroni correction was used to correct for multiple corrections in all cases, with a *p*-value of 0.001 (44 questions compared between groups).

This study was approved by the Institutional Review Board (IRB, or Ethics Committee) of Rutgers, the State University of New Jersey (IRB protocol code: 2022000237 and date of approval: 16 February 2022).

## 3. Results

### 3.1. Demographics

This population consisted of 269 NJ CTE teachers. For details on the demographics of this paper, please look at our previously published study and consult the Supplement [25] (Supplemental Table S1).

### 3.2. Vaccination Status by Study Factors

Most study participants (n = 244, 90.7%) had received the COVID-19 vaccination at the time of the study, while 19 (7.1%) did not receive the vaccine (Table 1). Those who had previously received a positive COVID-19 diagnosis were less likely to get the COVID-19 vaccine compared to those who did not receive a positive diagnosis (83.8% vs. 96.8%; *p* < 0.001) (Supplemental Table S2). The study found that those who worked in Cumberland or Ocean counties were more likely to not receive the COVID-19 vaccine when compared to those who worked in other counties (94.6% vs. 83.3%; *p* = 0.03) (Supplemental Table S2). Gender, race, age, NJ teaching experience, and overall teaching experiences were not associated with self-reported vaccination status among study participants (Supplemental Table S2). Those who were vaccinated were slightly older than those who were not vaccinated (mean age 46.4 vs. 44.1; *p* = 0.36, ANOVA test).

Of those who received the COVID-19 vaccine, 6.5% received a vaccine that only required one dose, whether that be Johnson and Johnson or another international vaccine. Most (93.5%) received a vaccine that required two doses, whether that be Moderna, Pfizer, or another international vaccine (Table 1). COVID-19 diagnosis, overall, or NJ teaching experience, age, gender, race, or county of employment were not associated with the number of doses one received (Supplemental Table S2).

Of those who have been vaccinated, 167 (77.7%) participants had received at least one COVID-19 booster vaccination at the time of this survey, and 48 (22.3%) had not (Table 1). Those who had not received a positive COVID-19 diagnosis had higher rates of getting the booster than those who had a previous positive diagnosis (86.7% vs. 65.2%, respectively; *p* < 0.001) (Supplemental Table S2). Males were more likely to get a booster when compared to females (86.3% vs. 77.1%, respectively; *p* = 0.05) (Supplemental Table S2). There were no differences in booster status by teaching experience (overall or NJ), age, race, or county. The mean age of those who received at least one booster was 47.0 years old (SD, 9.8), and those who did not receive the booster were, on average, 44.4 years old (SD, 9.1) (*p* = 0.11, ANOVA).

### 3.3. Perceptions of the Safety and Ease of Access to the Currently Available COVID-19 Vaccination

The survey asked how safe one believed the vaccine was before and after taking the vaccine; a total of 211 participants answered both questions. After receiving the vaccine, 3.8% of the participants believed that the vaccine was less safe than what they believed before they received the vaccine; 2.4% of participants believed that the vaccine was not safe at all before or after getting the vaccine. Overall, about half (48.3%) of participants believed that the vaccine was very safe both before and after getting the vaccine (Table 2). When stratified by COVID diagnosis, those who had a positive diagnosis were borderline more likely to believe that the vaccine was not safe at all when compared to those who did not previously receive a positive diagnosis (7.6% vs. 0.08%; *p* = 0.09) (Supplemental Table S2). There were no differences in perceptions of vaccine safety by COVID-19 diagnosis, overall and NJ teaching information, age, race, gender, and county of employment.

About 65.1% of all participants found that it was easy to get or would have been easy to get the vaccine for themselves (Table 1). Ease of vaccination was not different by number of doses, COVID-19 diagnosis, age, teaching experience (both NJ and Overall), race, or county of employment (Supplemental Table S2). There was a difference by vaccination status, as 61.1% of participants who did not get vaccinated found it very easy to get the vaccine, whereas only 26.5% of participants who received the vaccine found it very easy (*p* < 0.001). A greater proportion of participants who did not get the booster (47.9%) found it, or would have found it, very easy to get an initial COVID-19 vaccine compared to those who had received a booster (20.4%; *p* = 0.002). After getting the vaccine, men were significantly more likely to believe that it was somewhat easy to get the vaccine (30.8% female vs. 47.9% male), while women were more likely to believe that it was somewhat difficult (32.9% female vs. 16.9% male; *p* = 0.02) (Supplemental Table S2). The mean age of someone who found it very easy to get the vaccine was 45.5 (SD, 9.3); those who found it moderately easy were, on average, 45.6 (SD, 8.7), and those who found it very difficult were 48.2 (SD, 10.7). Using the ANOVA test, the *p*-value was 0.45.

### 3.4. Difficulty in Receiving the COVID-19 Vaccine

Several factors made it difficult for participants to get the vaccine. Overall, the three most frequent difficulties were: “It was difficult to find or make a vaccine appointment” (49.8%), “The waiting time for the vaccine was too long” (13.3%), and “That they were not eligible to get the vaccine at the time they tried” (12.9%). However, no one stated that transportation was a difficulty (Table 1). There were no significant differences in any of the obstacles when looking at the other factors (COVID-19 diagnosis, age, gender, overall teaching experience, and NJ teaching experience) and no difference in the rankings.

When data were stratified by vaccination status, only the reported difficulty to make an appointment was a significant factor for people to get the vaccine or not (*p* < 0.001). For participants who were vaccinated, the top three difficulty factors were the same as the overall group; however, for those who did not get vaccinated, the top choices were unspecified “other” (33.3%) or “not sure” (33.3%). This is expected as this group elected not to get the vaccine.

Those who received one dose were more likely to report the reason they did not get the vaccine was because they were “too busy to get vaccinated” when compared to those who got two doses (21.4% vs. 4.5%; *p* = 0.03). For those who received two doses, the top three obstacles were the same as the overall population, but for those who received one dose, the top three obstacles were: “It was difficult to find or make an appointment” (42.9%), “The waiting time was too long” (21.4%), and “I didn’t know where to get vaccinated” (14.3%).

When stratified by booster status, those who never received a booster were more likely to choose that a lack of childcare was a reason why getting the vaccine was difficult (1.8%), whereas 8.3% of those who did not get the booster chose the same reason for not getting it. There was no difference in the ranking of obstacle difficulties when stratified by booster status.

When stratified by race, there was no difference in the top three factors compared to the overall group. However, there was a difference in the response for the factor “I didn’t know where to go to get vaccinated” (4.0% NHW vs. 12.8% Other; *p* = 0.05).

When stratified by counties that one worked in, the study found that those who did not work in Cumberland or Ocean counties found that the wait time was too long (15.1%), while no one in Cumberland or Ocean counties specified this (*p* = 0.05).

### 3.5. Factors Pertaining to Confidence in COVID-19 Vaccines

Overall, the three most important factors that one wanted to know to increase confidence in the COVID-19 vaccine were: “My risk of getting sick with COVID-19 is bigger than the risk of side effects from the vaccine” (49.8%), “The vaccine works in protecting me from COVID-19” (46.4%), and “Agencies approving the vaccine are following strict rules” (34.8%) (Table 1).

When stratified by vaccination status, the study found that those who were vaccinated were more likely to believe that agencies approving the vaccine were following strict rules (36.7% vaccinated vs. 11.1% unvaccinated; *p* = 0.04) (Table 3). A difference was noted for the factor “Health agencies and WHO recommend the vaccine and agree it is safe” (34.4% vaccinated vs. 11.1% unvaccinated; *p* = 0.06). For those who were vaccinated, the ranking of what is most important stayed the same, while for those who were not vaccinated, the top three factors varied (Supplemental Table S3).

When stratified by vaccine dose number, the study found that those who received two doses were more likely to believe that health agencies and WHO recommend the vaccine and agree it is safe (7.1% one dose vs. 36.3% two doses; *p* = 0.04). For two doses, the ranking was the same as the overall group. For one dose, the ranking replaces “Agencies approving the vaccines are following strict rules” with “The vaccine cannot cause any immediate or long-term injury” as well as “The vaccine works in stopping the transmission from one person to another” (35.7% each).

When stratified by booster status, the study found that those who received a booster dose believed that the risk of getting sick with COVID-19 was greater than the risk of side effects from the vaccine (56.3% booster vs. 35.4% no booster; *p* = 0.01), the vaccine works in protecting against COVID-19 (53.9% booster vs. 35.4% no booster; *p* = 0.03), that the vaccine works in stopping the transmission from one person to another (38.3% booster vs. 18.8% no booster; *p* = 0.02), and that health agencies and WHO recommend the vaccine and agree it is safe (39.5% booster vs. 16.7% no booster; *p* = 0.003) (Table 3). For those who received the booster, the ranking varied from the overall group; however, for those who did not get a booster, the rank was the same as the overall group (Supplemental Table S3).

When stratified by COVID-19 diagnosis, those who did not have a positive COVID-19 diagnosis were significantly more likely to state that the vaccine works in protecting one from COVID-19 (54.8% no positive diagnosis vs. 37.7% positive diagnosis; *p* = 0.01) (Table 3). For those with a positive diagnosis, the ranks did not change from the overall group, but the rankings varied for those without a positive diagnosis (Supplemental Table S3).

There was a difference in age in the likeliness to report that the speed at which the vaccine was produced could compromise the safety of the vaccine. Those who believed so were, on average, 44.4 years old (SD, 9.3), and those who did not were, on average, 47.0 years old (SD, 9.6; *p* = 0.05). Choosing that the vaccine works in protecting against COVID-19 as an important factor also differed by overall teaching experience, with those who agreed, having, on average, 17.3 years of experience (SD, 7.6) and those who did not, having, on average, 45.5 years of experience (SD, 9.7; *p* = 0.05).

Women were more likely to believe that the risk of getting sick with COVID-19 was greater than the risk of side effects from the vaccine (57.1% female vs. 41.7% male; *p* = 0.04) (Table 3). For both genders, the ranking was the same as the overall group (Supplemental Table S3).

When stratified by race, the rankings were the same as the overall group (Supplemental Table S3). It should be noted that there was a difference between those who were NHW and those who were not when asked if they needed more information to get the COVID-19 vaccine (9.2% NHW vs. 20.5% other; *p* = 0.05) (Table 3).

### 3.6. Important Factors Regarding Receiving the COVID-19 Vaccine

The three most important factors for one to be more likely to receive the COVID-19 vaccine were: living life without restrictions (45.1%), equal access to the vaccine regardless of income or race (32.2%), and the school district or school required the vaccine (25.3%) (Table 1).

When stratified by vaccination status, the study found that those who were not vaccinated found that the factor that was most important to them was that they will be free to choose to get the vaccine or not with no consequences (55.6%), which was significantly different than those who were vaccinated (18.6%) with a *p*-value of 0.001. The rank for those who have been vaccinated was the same as the overall rank (Supplemental Table S4).

When stratified by the number of doses, only the fact that pharmaceutical companies will not make large profits was significantly different (35.7% one dose vs. 12.9% two doses; *p* = 0.03). The ranking for both one and two doses is the same as the overall group.

When stratified by booster status, those who received the booster were more likely to say that the fact that everybody will have equal access to the vaccine regardless of income or race (39.5% booster vs. 16.7% no booster; *p* = 0.003) was important to them. Those who did not receive the booster were more likely to say that the factor that “I will be free to choose if I get the vaccine or not with no consequences” (15.0% booster vs. 31.3% no booster; *p* = 0.02), and that “The school district or school that I work for required vaccination” (23.4% booster vs. 39.6% no booster; *p* = 0.04) were most important to them (Table 4).

When stratified by COVID-19 diagnosis, those who had a positive diagnosis believed they would be free to choose if they get the vaccine or not with no consequences was significantly more important to them when compared to those with no positive diagnosis (32.1% positive diagnosis vs. 12.9% non-positive diagnosis; *p* < 0.001). For those with a positive diagnosis, the top three factors were: “Once vaccinated, I will be able to live my life with no restrictions” (42.5%), “I will be free to choose if I get the vaccine or not with no consequences” (32.1%), and “Everybody will have equal access to the vaccine regardless of income or race” (29.2%). The ranking for those with no positive diagnosis was the same as the overall ranking.

This study suggested a difference as one gains more overall experience in teaching if one received the vaccine because it was required by the school. The study suggested an overall difference between the average amount of overall experience a teacher who was vaccinated because their school required it was 14.6 years (SD, 7.1) and those who did not select this answer option was 16.9 years (SD, 7.1; *p* = 0.04). The average number of teaching experience years overall also differed when deciding if everyone having equal access to the vaccine was important. Those who did had a mean number of 17.9 years (SD, 8.1) and those who did not had 15.5 years (SD, 7.2; *p* = 0.03). The same was true for teaching experience in NJ as well, with those who agreed having a mean experience of 16.8 years (SD, 7.5) and those who did not, 14.7 years (SD, 6.8; *p* = 0.04). Teaching experience in NJ also differed when asked if one will be free to choose if they get the vaccine without consequences was important to them. Those who agreed had a mean of 13.5 years (SD, 6.5) and those who did not had 15.9 years (SD, 7.2; *p* = 0.03).

When stratified by race, there was a difference with the factor “I will be free to choose if I get the vaccine or not with no consequences” (16.7% NHW vs. 41.0% other; *p* = 0.0002) (Table 4). The ranking of factors for those who identify as NHW had the same ranking as the overall group; however, the ranking of those who do not identify as NHW varied (Supplemental Table S4).

When stratified by county the participants were employed in, for those who did work in Cumberland or Ocean counties, an important factor in whether or not to get the vaccine was the belief that once they were vaccinated, then, they were able to live their life with no restrictions (66.7% Cumberland and Ocean counties vs. 42.9% other; *p* = 0.03). Two other important factors were: “Everybody will have equal access to the vaccine regardless of race or income” (20.8% Cumberland and Ocean vs. 34.1% other counties; *p* = 0.25) (Table 4), and “There are no other reasons why so many people are sick” (12.5% Cumberland and Ocean vs. 3.4% other counties; *p* = 0.07) (Supplemental Table S4).

### 3.7. COVID-19 Case Information-Study Participants

Approximately 46.1% of participants have had COVID-19, while 45.7% did not have COVID-19 in the past, and 8.3% did not know if they had COVID-19 (Table 1). Of those who had a positive COVID-19 diagnosis (n = 106, 46.1%), 60.4% did not seek medical care, 34.9% received medical care but were not hospitalized, and 0.9% were hospitalized (Table 1). For both questions, the number of doses, overall teaching experience, gender, race, and county were not significantly different between those who had a positive diagnosis and those who did not receive a positive diagnosis of COVID-19 at the time the survey was completed (Supplemental Table S5). Of those who have received the vaccine, 42.8% had a positive COVID-19 diagnosis, while 77.8% of people who did not receive the vaccine were diagnosed (*p* = 0.02). There was no significant difference in the level of care when stratified by vaccination status (Table 5). Of those who received the booster, 35.9% had a positive COVID-19 diagnosis, while 66.7% of people who did get the booster were diagnosed (*p* < 0.001). There was no significant difference in the level of care when stratified by vaccination status (Table 5).

### 3.8. COVID-19 Case Information-Family Members

Among study participants, 12.5% had family members older than 70 years living in their home, 66.4% had family older than 70 not living in their home, and 21.1% did not have family over 70 (Table 1).

Approximately three in five (61.0%) participants have had someone close to them (family close friends or community networks) become seriously ill or die because of COVID-19 (Table 1). For both questions, there were no differences by number of doses received, booster status, COVID-19 diagnosis, age, gender, race, or county of work had any significant bearing (Supplemental Table S5). For participants who were vaccinated, 13.5% had a family member older than 70 that was living with them, and none of those who were not vaccinated had someone living with them (*p* = 0.09) (Supplemental Table S5).

The overall mean of those who knew anyone close to them who had a serious case of COVID-19 (mean, 17.2; SD, 7.5) was higher than those who did not (mean, 15.2; SD, 7.5; *p* = 0.04).

## 4. Discussion

This study provides valuable insights regarding COVID-19 vaccine hesitancy among NJ teachers. This survey allowed teachers to indicate what factors made it difficult to get the COVID-19 vaccine, factors that were important to increase vaccine confidence, and factors that made it more likely they would get the vaccine. This study had a number of findings that are important to highlight: the first being that those who had been diagnosed with COVID-19 were less likely to get the COVID-19 vaccine or the booster dose during the initial stages of the pandemic—this is consistent with previous studies [26]. We also found that women were less likely to get the booster dose of the vaccine, which is consistent with the previous literature [19]. In our study, we found that 58.8% of teachers who were not vaccinated were female, while other studies found that 90.3% of the unvaccinated study population (consisting of healthcare workers) identified as females [27]. The difference might be due to their profession or the location of the study. A previous study explored the relationship between political partisanship and the COVID-19 vaccination rate [28]. However, we cannot draw any similar conclusions as we do not know the political affiliations of any of our participants, and the counties we chose to stratify (Ocean and Cumberland) do not lean towards the same political party [29].

Regarding access to the COVID-19 vaccine, those who chose not to get the vaccine thought that it would have been very easy to get the vaccine, while those who did get the vaccine found it to be more difficult; the same observations were reported regarding the booster dose. This might have been a matter of perception, as in 2021, there were longer wait times associated with people obtaining the COVID-19 vaccine [30]. This is again echoed in the most frequent difficulty of getting vaccinated, “It was difficult to find or make a vaccine appointment”, which was almost four times more likely to be chosen than the difficulty, “The waiting time for the vaccine was too long.” There was also a difference between people who did and did not identify as NHW when it came to knowing where to get the vaccine. Those who did not identify as NHW identified this problem more than those who identified as NHW; this could suggest that this group had a harder time finding vaccine locations. This is consistent with other studies that have found a threefold disparity between vaccination rates and race, in part due to access and availability [19,31,32,33]. Those who do not identify as NHW, they were also twice as likely to say that an important factor for them to get the COVID-19 vaccine was that they would be free to choose whether or not to get the vaccine without consequences. While there has been a study about vaccine freedom in Italy, the study did not examine any relationship between hesitancy and race [34].

In the summer of 2023, there was an increase in COVID-19 cases and subsequently hospitalization and death rates [35]. This is resulting in some schools asking students, staff, and visitors to wear a mask on campus [35]. As of May 2023, the CDC has stated about 17% of the U.S. population has the updated (bivalent) booster dose [34]. Since there is a link between the booster dose and lower cases of hospitalization [36], it is possible a lower rate of booster dose receipt can contribute to higher hospitalization rates. It is possible that increasing the booster dose uptake can help decrease hospitalization and, thus, vaccine education is still an important factor.

Vaccine hesitancy can be analyzed through the lens of the Health Belief Model (HBM), which is a theoretical framework used to predict health-related behaviors based on an individual or group’s beliefs and behaviors [37]. The model includes six constructs, such as the perceived benefits of the health action (getting the COVID-19 vaccine) and its perceived susceptibility and severity. The model also includes constructs like perceived barriers to taking the health action, along with self-efficacy and cues to action [37,38,39]. In this study, we looked at the perceived benefits of getting the vaccine (“The vaccine works in protecting me from COVID-19” and “The vaccine works in stopping the transmission from one person to another”). In both of these factors, those who chose to get vaccinated and get the booster dose were more likely to state these factors were important to them to get the vaccine. A literature review found that those who saw large benefits in the vaccine would be more likely to get the vaccine [39]. The present study also found that barriers such as vaccine appointment availability were a factor when it came to getting vaccinated. This does differ from HBM because, as discussed above, those who perceived the barrier as higher were more likely to get the vaccine. However, as stated above, we did find participants who have had COVID-19 in the past were less likely to receive the vaccine. If people think having had COVID-19 is an equivalent or better protector against getting COVID-19 again, then they might perceive the severity of the disease or their susceptibility to getting infected again as lower. This is consistent with literature reviews, which have also found lower rates of perceived susceptibility and severity correlated with vaccine hesitancy [39]. The present study also looks at the construct call for action in asking if an important reason to get vaccinated was if there was a school requirement. We found 27.0% of participants reported this, exemplifying the call for action construct. The present study, however, did not explore anything with the construct of self-efficacy.

For strengths and limitations for this study please reference our previously published study [25].

## 5. Conclusions

This study provides insights regarding COVID-19 vaccination and safety practices among secondary educational professionals across the state of NJ. When stratified by variables (vaccination status, booster status, COVID-19 diagnosis status, gender, race, and county of employment), data suggested differences in levels of COVID-19-related concerns and confidence in or importance of the vaccines available at the time in 2022. Overall, those who had not received the vaccine at the time of the survey were more likely to have reservations about the vaccine and were less concerned about contracting COVID-19. Those who had a positive COVID-19 diagnosis were less likely to receive the COVID-19 vaccine and the booster vaccine. Most participants across gender and racial/ethnic backgrounds reported they have sought out vaccination opportunities; however, there were differences in which groups remained concerned about different factors, including side effects of the vaccine, and sub-groups reported varied factors of importance to them.

Besides future research with a larger sub-population to represent the general population and not only secondary educational professionals, this study suggests the general NJ population, particularly those working in Cumberland or Ocean Counties, would benefit from a further distribution of vaccine education efforts and work to mitigate concerns about the efficacy and safety of the vaccine. As the COVID-19 pandemic continues to progress, educational outreach efforts will play a large role in convincing NJ citizens to receive the vaccine, boosters, and practice other disease-limiting behaviors. Since this survey was administrated in 2022, a follow-up study could focus on how COVID-19 vaccination rates have changed both between and within the counties in the state of NJ, along with other U.S states.

## Figures and Tables

**Table 1:** Frequency regarding COVID-19 vaccine information among New Jersey secondary and high school teachers based on survey data.

	N (n = 269)	% of Total	% of Answered
Have you received a COVID-19 vaccine?			
Yes	244	90.7%	90.7%
No	19	7.1%	7.1%
IPNA	6	2.2%	2.2%
Missing	0	0.0%	--
Did you receive a vaccine product that requires only one dose or two dose? (n = 250)			
One dose	14	5.6%	6.5%
Two doses	201	80.4%	93.5%
I don’t know	0	0.0%	0.0%
IPNA	0	0.0%	0.0%
Missing	35	14.0%	--
Have you received a COVID-19 booster vaccine? (n = 250)			
Yes	167	66.8%	77.7%
No	48	19.2%	22.3%
IPNA	0	0.0%	0.0%
Missing	35	14.0%	--
How safe do you think a COVID-19 vaccine was before you got the vaccine? (n = 250)			
Not at all safe	8	3.2%	3.8%
A little safe	21	8.4%	10.0%
Moderately safe	76	30.4%	36.0%
Very safe	106	42.4%	50.2%
IPNA	0	0.0%	0.0%
Missing	39	15.6%	--
How safe do you think a COVID-19 vaccine was after you got the vaccine? (n = 250)			
Not at all safe	7	2.8%	3.3%
A little safe	15	6.0%	7.1%
Moderately safe	67	26.8%	31.8%
Very safe	122	48.8%	57.8%
IPNA	0	0.0%	0.0%
Missing	39	15.6%	--
How easy was it to get, or would it have been to get, a COVID-19 vaccine for yourself?			
Very easy	68	25.3%	29.3%
Somewhat easy	83	30.9%	35.8%
Somewhat difficult	62	23.0%	26.7%
Very difficult	17	6.3%	7.3%
Not sure	2	0.7%	0.9%
IPNA	0	0.0%	0.0%
Missing	37	13.8%	--
What made it difficult, or would have made it difficult, for you to get a COVID-19 vaccine? (Check all that apply)			
I didn’t have time off work	23	8.6%	9.9%
I didn’t have transportation	0	0.0%	0.0%
I didn’t know where to go to get the vaccine	12	4.5%	5.2%
I was too busy	13	4.8%	5.6%
I wasn’t eligible to get a COVID-19 vaccine	30	11.2%	12.9%
It was difficult to arrange for childcare	8	3.0%	3.4%
It was difficult to find or make an appointment	116	43.1%	49.8%
It’s too far away	11	4.1%	4.7%
The hours of operation are inconvenient	18	6.7%	7.7%
The waiting time was too long	31	11.5%	13.3%
Other	18	6.7%	7.7%
Not Sure	15	5.6%	6.4%
IPNA	8	3.0%	3.4%
Missing	36	13.4%	--
What was important for you to know to make you more confident in the COVID-19 vaccine? (Check all that apply)			
Agencies approving the vaccines are following strict rules	81	30.1%	34.8%
Health agencies and WHO recommend the vaccine and agree it is same	76	28.3%	32.6%
It is impossible to get COVID-19 or any other disease from the vaccine itself or its components	40	14.9%	17.2%
My risk of getting sick with COVID-19 is bigger than the risk of side effects from the vaccine	116	43.1%	49.8%
The fast production of the vaccine did not compromise its safety	71	26.4%	30.5%
The vaccine cannot cause any immediate or long-term injury	78	29.0%	33.5%
The vaccine works in protecting me from COVID-19	108	40.1%	46.4%
The vaccine works in stopping the transmission from one person to another	74	27.5%	31.8%
I do not need any other information	25	9.3%	10.7%
Other	10	3.7%	4.3%
IPNA	8	3.0%	3.4%
Missing	36	13.4%	--
What else was important for you to know that made you more likely to take the COVID-19 vaccine? (Check all that apply)			
Everybody will have equal access to the vaccine regardless of income or race	75	27.9%	32.2%
I will be free to choose if I get the vaccine or not with no consequences	50	18.6%	21.5%
Once vaccinated I will be able to live my life with no restrictions	105	39.0%	45.1%
Pharmaceutical companies will not make large profits from the vaccine	33	12.3%	14.2%
The school district or school I work for required the vaccine	59	21.9%	25.3%
There are no other reasons why so many people are sick	10	3.7%	4.3%
Those with concerns about the vaccine have opportunities to share their opinions with the public	13	4.8%	5.6%
IPNA	10	3.7%	4.3%
Missing	36	13.4%	--
Do you have any close family members older than 70 years?			
Yes, living together	29	10.8%	12.5%
Yes, not living together	154	57.2%	66.4%
No	49	18.2%	21.1%
IPNA/Missing	37	13.8%	--
Do you personally know anyone in your family, group of friends, or community networks who became seriously ill or died as a result of COVID-19?			
Yes	141	52.4%	61.0%
No	90	33.5%	39.0%
IPNA/Missing	38	14.1%	--
To your knowledge, do you have or have you had COVID-19?			
Yes, living together	106	39.4%	46.1%
No	105	39.0%	45.7%
I do not know	19	7.1%	8.3%
IPNA/Missing	39	14.5%	--
Describe the level of care you received, or are receiving (n = 106)			
Did not seek medical care	64	60.4%	62.7%
Received medical care but was not hospitalized	37	34.9%	36.3%
IPNA/Missing	4	3.8%	--

Note: IPNA is “I prefer not to answer”. Two participants were not sure how hard it was or would be to get the COVID-19 vaccine. Only two participants chose “I couldn’t go on my own (I have a physical limitation)”.

**Table 2:** Perception of how safe the vaccine is before and after getting the vaccine among NJ secondary and high school teachers.

	How Safe Do You Think a COVID-19 Vaccine Was before You Got the Vaccine?								
How safe do you think a COVID-19 vaccine was after you got the vaccine?		**Not at All Safe**	**%**	**A Little Safe**	**%**	**Moderately Safe**	**%**	**Very Safe**	**%**
	Not at all safe	5	2.4%	1	0.5%	1	0.5%	0	0.0%
	A little safe	2	0.9%	11	5.2%	2	0.9%	0	0.0%
	Moderately safe	1	0.5%	7	3.3%	55	26.1%	4	1.9%
	Very safe	0	0.0%	2	0.9%	18	8.5%	102	48.3%

Note: Percentage is the percentage of total table count.

**Table 3:** Factors pertaining to COVID-19 vaccine confidence among New Jersey secondary and high school teachers based on survey data.

What Was Important for You to Know to Make You More Confident in the COVID-19 Vaccine? (Check All That Apply)							
**Vaccination Status**	**Vaccinated (n = 215)**	**%**	**Not Vaccinated or IPNA (n = 18)**	**%**	**Fisher’s Exact Test**	**Total**	**%**
Agencies approving the vaccines are following strict rules	79	36.7%	2	11.1%	0.04 *	81	34.8%
My risk of getting sick with COVID-19 is bigger than the risk of side effects from the vaccine	111	51.6%	5	27.8%	0.08	116	49.8%
**Booster Status**	**Taken (n = 167)**	**%**	**Not Taken (n = 48)**	**%**	**Fisher’s Exact Test**	**Total**	**%**
Agencies approving the vaccines are following strict rules	63	37.7%	16	33.3%	0.61	79	36.7%
Health agencies and WHO recommend the vaccine and agree it is the same	66	39.5%	8	16.7%	0.003 **	74	34.4%
My risk of getting sick with COVID-19 is bigger than the risk of side effects from the vaccine	94	56.3%	17	35.4%	0.01 *	111	51.6%
The vaccine works in protecting me from COVID-19	90	53.9%	17	35.4%	0.03 *	107	49.8%
The vaccine works in stopping the transmission from one person to another	64	38.3%	9	18.8%	0.02 *	73	34.0%
**COVID-19 Diagnosis**	**Positive (n = 106)**	**%**	**Not Positive (n = 124)**	**%**	**Fisher’s Exact Test**	**Total**	**%**
Agencies approving the vaccines are following strict rules	40	37.7%	41	33.1%	0.49	81	35.2%
My risk of getting sick with COVID-19 is bigger than the risk of side effects from the vaccine	49	46.2%	65	52.4%	0.36	114	49.6%
The vaccine works in protecting me from COVID-19	40	37.7%	68	54.8%	0.01 **	108	47.0%
The vaccine works in stopping the transmission from one person to another	28	26.4%	46	37.1%	0.09	74	32.2%
**Gender**	**Female (n = 147)**	**%**	**Male (n = 72)**	**%**	**Fisher’s Exact Test**	**Total**	**%**
Agencies approving the vaccines are following strict rules	53	36.1%	27	37.5%	0.88	80	36.5%
My risk of getting sick with COVID-19 is bigger than the risk of side effects from the vaccine	84	57.1%	30	41.7%	0.04 *	114	52.1%
The vaccine works in stopping the transmission from one person to another	51	34.7%	21	29.2%	0.45	72	32.9%
**Race**	**Non-Hispanic White (n = 174)**	**%**	**Other (n = 39)**	**%**	**Fisher’s Exact Test**	**Total**	**%**
Agencies approving the vaccines are following strict rules	65	37.4%	13	33.3%	0.72	78	36.6%
My risk of getting sick with COVID-19 is bigger than the risk of side effects from the vaccine	95	54.6%	16	41.0%	0.16	111	52.1%
The vaccine works in stopping the transmission from one person to another	62	35.6%	9	23.1%	0.19	71	33.3%
I do not need any other information	16	9.2%	8	20.5%	0.05 *	24	11.3%
**Counties**	**Cumberland and Ocean (n = 24)**	**%**	**Other Counties (n = 205)**	**%**	**Fisher’s Exact Test**	**Total**	**%**
Agencies approving the vaccines are following strict rules	8	33.3%	73	35.6%	1.00	81	35.4%
My risk of getting sick with COVID-19 is bigger than the risk of side effects from the vaccine	12	50.0%	104	50.7%	1.00	116	50.7%
The vaccine works in stopping the transmission from one person to another	5	20.8%	69	33.7%	0.25	74	32.3%

Note: IPNA is “I prefer not to answer” * *p* < 0.05, ** *p* < 0.01.

**Table 4:** Important factors regarding the COVID-19 vaccine among New Jersey secondary and high school teachers based on survey data.

What Else Was Important for You to Know That Made You More Likely to Take the COVID-19 Vaccine? (Check All That Apply)							
**Booster Status**	**Taken (n = 167)**	**%**	**Not Taken (n = 48)**	**%**	**Fisher’s Exact Test**	**Total**	**%**
Everybody will have equal access to the vaccine regardless of income or race	66	39.5%	8	16.7%	0.003 **	74	34.4%
I will be free to choose if I get the vaccine or not with no consequences	25	15.0%	15	31.3%	0.02 *	40	18.6%
Once vaccinated I will be able to live my life with no restrictions	84	50.3%	19	39.6%	0.25	103	47.9%
The school district or school I work for required the vaccine	39	23.4%	19	39.6%	0.04 *	58	27.0%
**Gender**	**Female (n = 147)**	**%**	**Male (n = 72)**	**%**	**Fisher’s Exact Test**	**Total**	**%**
Everybody will have equal access to the vaccine regardless of income or race	51	34.7%	23	31.9%	0.76	74	33.8%
Once vaccinated, I will be able to live my life with no restrictions	63	42.9%	39	54.2%	0.15	102	46.6%
The school district or school I work for required the vaccine	35	23.8%	21	29.2%	0.41	56	25.6%
**Race**	**Non-Hispanic White (n = 174)**	**%**	**Other (n = 39)**	**%**	**Fisher’s Exact Test**	**Total**	**%**
Everybody will have equal access to the vaccine regardless of income or race	61	35.1%	12	30.8%	0.71	73	34.3%
I will be free to choose if I get the vaccine or not with no consequences	29	16.7%	16	41.0%	0.002 **	45	21.1%
Once vaccinated, I will be able to live my life with no restrictions	84	48.3%	15	38.5%	0.29	99	46.5%
The school district or school I work for required the vaccine	46	26.4%	9	23.1%	0.84	55	25.8%
**Counties**	**Cumberland and Ocean (n = 24)**	**%**	**Other Counties (n = 205)**	**%**	**Fisher’s Exact Test**	**Total**	**%**
Everybody will have equal access to the vaccine regardless of income or race	5	20.8%	70	34.1%	0.25	75	32.8%
Once vaccinated, I will be able to live my life with no restrictions	16	66.7%	88	42.9%	0.03 *	104	45.4%
The school district or school I work for required the vaccine	6	25.0%	52	25.4%	1.00	58	25.3%

Note: IPNA is “I prefer not to answer” * *p* < 0.05, ** *p* < 0.01.

**Table 5:** COVID-19 case information among New Jersey secondary and high school teachers based on survey data.

To Your Knowledge, Do You Have or Have You Had COVID-19?							
**Vaccination Status**	**Vaccinated (n = 215)**	**%**	**Not Vaccinated or IPNA (n = 18)**	**%**	**Fisher’s Exact Test**	**Total**	**%**
Yes	92	42.8%	14	77.8%	0.02 *	106	45.5%
No	102	47.4%	3	16.7%		105	45.1%
**Booster Status**	**Taken (n = 167)**	**%**	**Not Taken (n = 48)**	**%**	**Fisher’s Exact Test**	**Total**	**%**
Yes	60	35.9%	32	66.7%	<0.001 ***	92	42.8%
No	88	52.7%	14	29.2%		102	47.4%
I do not know	16	9.6%	2	4.2%		18	8.4%
**Number of Vaccine Doses Received**	**One dose (n = 14)**	**%**	**Two doses (n = 201)**	**%**	**Fisher’s Exact Test**	**Total**	**%**
Yes	7	50.0%	85	42.3%	0.10	92	42.8%
No	4	28.6%	98	48.8%		102	47.4%
I do not know	3	21.4%	15	7.5%		18	8.4%
**Gender**	**Female (n = 147)**	**%**	**Male (n = 72)**	**%**	**Fisher’s Exact Test**	**Total**	**%**
Yes	68	46.3%	30	41.7%	0.59	98	44.7%
No	66	44.9%	35	48.6%		101	46.1%
I don’t know	10	6.8%	7	9.7%		17	7.8%
**Race**	**Non-Hispanic White (n = 174)**	**%**	**Other (n = 39)**	**%**	**Fisher’s Exact Test**	**Total**	**%**
Yes	77	44.2%	16	41.0%	0.93	93	43.7%
No	81	46.6%	20	51.2%		101	47.4%
I don’t know	13	7.5%	3	7.7%		16	7.5%
**County**	**Cumberland and Ocean (n = 24)**	**%**	**Other Counties (n = 205)**	**%**	**Fisher’s Exact Test**	**Total**	**%**
Yes	14	58.3%	90	43.9%	0.48	104	45.4%
No	9	37.5%	94	45.9%		103	45.0%

Note: IPNA is “I prefer not to answer”. * *p* < 0.05, *** *p* < 0.001; one unvaccinated participant did not know if they had a positive COVID-19 diagnosis; one participant from Cumberland and Ocean did not know if they had had a positive COVID-19 diagnosis.

## Data Availability

Data were obtained via a survey titled “COVID-19 Vaccine Survey for NJ teachers 2022”, which was delivered virtually to individuals who completed work-based learning (WBL) training provided by the NJSS between 2014 and 2022.

## Supplementary Materials

The following supporting information can be downloaded at: https://www.mdpi.com/article/10.3390/vaccines11111667/s1, Table S1: Demographics of the study sample; Table S2: Vaccination status information among New Jersey secondary and high school teachers based on survey data; Table S3: Factors pertaining to COVID-19 vaccine confidence among New Jersey secondary and high school teachers based on survey data; Table S4: Important factors regarding the COVID-19 vaccine among New Jersey secondary and high school teachers based on survey data; Table S5: COVID-19 case information among New Jersey secondary and high school teachers based of off survey data; full survey.
