# Supplementary material for: Factors Associated with COVID-19 Vaccine Hesitancy and Case Status among New Jersey Secondary Educational Professionals
Source: Vaccines (Basel). 2023 Oct 31;11(11):1667. doi: 10.3390/vaccines11111667 (PMC10674534; doi:10.3390/vaccines11111667)
Supplement: Supplementary file 1 [file vaccines-11-01667-s001.zip › vaccines-2603434-supplementary/vaccines-supplementary-Proofread.pdf]

**Supplemental Table S1**: Demographics of study sample			
	N (n=269)	% of Total	% Answered
School County region:			
North	100	37.2%	43.7%
Central	84	31.2%	36.7%
South	45	16.7%	19.7%
Missing	40	14.9%	
Race and Ethnicity[a]*:*			
American Indian or Alaskan Native	1	0.4%	0.4%
Native Hawaiian or Other Asian-Pacific Islander	0	0.0%	0.0%
Middle Eastern/North African	2	0.7%	0.9%
Hispanic Asian	1	0.4%	0.4%
Hispanic Black	4	1.5%	1.7%
Hispanic White	17	6.3%	7.4%
Non-Hispanic Asian	3	1.1%	1.3%
Non-Hispanic Black	10	3.7%	4.4%
Non-Hispanic White	175	65.1%	76.4%
I prefer not to answer this question	13	4.8%	5.7%
Other	9	3.3%	3.9%
Missing	40	14.9%	
Gender Identity			
Male	72	26.8%	31.4%
Female	148	55.0%	64.6%
Other/Multigender	3	1.1%	1.3%
I prefer not to answer	4	1.5%	1.7%
Missing	40	14.9%	
**Birth Year (Mean±SD, Kolmogorov–Smirnov, p-value)**	1976±9.6, 0.06		
Pre-1950-1959	11	4.1%	4.8%
1960-1969	49	18.2%	21.5%
1970-1979	81	30.1%	35.5%
1980-1989	70	26.0%	30.7%
1990-2000	17	6.3%	7.5%
Missing	41	15.2%	
**Number of Years Teaching in NJ (Mean±SD, Kolmogorov–Smirnov, p-value)**	15.4±7.1, 0.14		
**Number of Years of Teaching Overall (Mean±SD, Kolmogorov–Smirnov, p-value)**	16.2±7.4, 0.08		
What is the highest education degree completed?			
Bachelor's degree	59	21.9%	25.8%
Master's degree	146	54.3%	63.8%
Doctoral degree	6	2.2%	2.6%
Other	18	6.7%	7.9%
Missing/NA/I prefer not to answer	40	14.9%	
**How many years of post-secondary education (after high school) have you completed? (Mean and SD)**	6.4±2.6		

[a] Participants had the option to choose multiple race categories.

Supplemental Table S2: Vaccination status information among New Jersey secondary and high school teachers based on Survey Data

COVID-19 Diagnosis	Positive (n=106)	%	Not Positive (n=124)	%	Fisher's Exact Test	Total	%
Have you received a COVID-19 vaccine?							
Yes	92	86.8%	120	96.8%	<0.001***	212	92.2%
No	14	13.2%	4	3.2%		18	7.8%
Did you receive a vaccine requiring only one dose or two doses? (n=212)							
One dose	7	7.6%	7	5.8%	0.78	14	6.6%
Two doses	85	92.4%	113	94.2%		198	93.4%
I don't know	0	0.0%	0	0.0%		0	0.0%
Have you received a COVID-19 booster vaccine (n=212)							
Yes	60	65.2%	104	86.7%	<0.001***	164	77.4%
No	32	34.8%	16	13.3%		48	22.6%
How safe do you think a COVID-19 vaccine was before you got it? (n=212)							
Not at all safe	7	7.6%	1	0.8%	0.09	8	3.8%
A little safe	8	8.7%	13	10.8%		21	9.9%
Moderately safe	31	33.7%	45	37.5%		76	35.8%
Very safe	44	47.8%	59	49.2%		103	48.6%
How safe do you think a COVID-19 vaccine was after getting it? (n=212)							
Not at all safe	5	5.4%	2	1.7%	0.18	7	3.3%
A little safe	8	8.7%	7	5.8%		15	7.1%
Moderately safe	32	34.8%	35	29.2%		67	31.6%
Very safe	45	48.9%	74	61.7%		119	56.1%
How easy was it to get, or would it have been to get, a COVID-19 vaccine for yourself?							
Very easy	36	34.0%	32	25.8%	0.34	68	29.6%
Somewhat easy	37	34.9%	46	37.1%		83	36.1%
Somewhat difficult	25	23.6%	34	27.4%		59	25.7%
Very difficult	6	5.7%	11	8.9%		17	7.4%

Gender	Female (n=147)	%	Male (n=72)	%	Fisher's Exact Test	Total	%
Have you received a COVID-19 vaccine?							
Yes	139	94.6%	66	91.7%	0.53	205	93.6%
No	7	4.8%	5	6.9%		12	5.5%
Did you receive a vaccine product that requires only one dose or two doses? (n=205)							
One dose	8	5.6%	5	7.6%	0.76	13	6.3%
Two doses	131	94.2%	61	92.4%		192	93.7%
Have you received a COVID-19 booster vaccine? (n=205)							
Yes	103	74.1%	57	86.3%	0.05*	160	78.0%
No	36	25.8%	9	13.6%		45	22.0%
How safe do did you think a COVID-19 vaccine was before you got it? (n=205)							
Not at all safe	6	4.3%	2	3.0%	0.44	8	3.9%
A little safe	15	10.8%	4	6.0%		19	9.2%
Moderately safe	53	38.1%	22	33.3%		75	36.6%
Very safe	63	45.3%	38	57.6%		101	49.3%
How safe do you think a COVID-19 vaccine is after you got it? Would you say:(n=205)							
Not at all safe	5	3.6%	2	3.0%	0.28	7	3.4%
A little safe	11	7.9%	2	3.0%		13	6.3%
Moderately safe	48	34.5%	18	27.3%		66	32.2%
Very safe	73	45.3%	44	66.7%		117	57.1%
How easy was it to get, or would it have been to get, a COVID-19 vaccine for yourself?							
Very easy	40	27.4%	22	31.0%	0.02*	62	28.6%
Somewhat easy	45	30.8%	34	47.9%		79	36.4%
Somewhat difficult	48	32.9%	12	16.9%		60	27.7%
Very difficult	13	8.9%	3	4.2%		16	7.4%

Race	Non-Hispanic White (n=174)	%	Other (n=39)	%	Fisher's Exact Test	Total	%
Have you received a COVID-19 vaccine?							
Yes	166	95.4%	34	87.2%	0.07	200	93.9%
No	8	4.6%	5	12.8%		13	6.1%
Missing/NA/IPNA	0	0.0%	0	0.0%		0	0.0%
Did you receive a vaccine product that requires only one dose or two doses? (n=200)							
One dose	12	7.2%	1	2.9%	0.70	13	6.5%
Two doses	154	92.8%	33	97.1%		190	93.5%
Have you received a COVID-19 booster vaccine? (n=200)							
Yes	130	78.3%	28	82.4%	0.82	158	79.0%
No	36	21.7%	6	17.7%		42	21.0%
How safe do did you think a COVID-19 vaccine was before you got it? (n=200)							
Not at all safe	5	3.0%	3	8.8%	0.21	8	4.0%
A little safe	13	7.8%	5	14.7%		18	9.0%
Moderately safe	60	36.1%	12	35.3%		72	36.0%
Very safe	85	51.2%	14	41.2%		99	45.5%
How safe do you think a COVID-19 vaccine is after you got it? (n=200)							
Not at all safe	5	3.0%	2	5.9%	0.77	7	3.5%
A little safe	10	6.0%	2	5.9%		12	6.0%
Moderately safe	51	30.7%	12	35.2%		63	31.5%
Very safe	97	58.4%	18	52.9%		115	57.5%
How easy was it to get, or would it have been to get, a COVID-19 vaccine for yourself?							
Very easy	46	26.6%	14	35.6%	0.36	62	29.0%
Somewhat easy	61	35.3%	15	38.5%		76	35.5%
Somewhat difficult	52	30.1%	8	20.5%		61	28.5%
Very difficult	14	8.1%	1	2.6%		15	7.0%

Counties	Cumberland and Ocean (n=24)	%	Other Counties (n=205)	%	Fisher's Exact Test	Total	%
Have you received a COVID-19 vaccine?							
Yes	20	83.3%	194	94.6%	0.03*	214	93.4%
No	4	16.7%	8	3.9%		12	5.2%
Did you receive a vaccine product that requires only one dose or two doses? (n=214)							
One dose	3	15.0%	17	8.8%	0.13	20	8.7%
Two doses	11	55.0%	183	94.3%		194	84.7%
Have you received a COVID-19 booster vaccine? (n=214)							
Yes	16	80.0%	150	77.3%	1.00	166	77.6%
No	4	20.0%	44	22.7%		48	22.4%
How safe do did you think a COVID-19 vaccine was before you got it? (n=214)							
Not at all safe	1	5.0%	7	3.6%	0.52	8	3.7%
A little safe	1	5.0%	19	9.8%		20	9.3%
Moderately safe	5	25.0%	71	36.6%		76	35.5%
Very safe	13	65.0%	93	47.9%		106	49.5%
How safe do you think a COVID-19 vaccine is after you got it? (n=214)							
Not at all safe	0	0.0%	7	3.6%	0.23	7	3.3%
A little safe	2	10.0%	12	6.2%		14	6.5%
Moderately safe	3	15.0%	64	33.0%		67	31.3%
Very safe	15	75.0%	107	55.2%		122	57.0%
How easy was it to get, or would it have been to get, a COVID-19 vaccine for yourself?							
Very easy	10	41.7%	56	27.7%	0.31	66	29.2%
Somewhat easy	9	37.5%	72	35.6%		81	35.8%
Somewhat difficult	5	20.8%	57	28.2%		62	27.4%
Very difficult	0	0.0%	17	8.4%		17	7.5%

Note: IPNA is "I prefer not to answer". *p=<0.05, ***p=<0.001.

**Supplemental Table S3**: Factors pertaining to COVID-19 vaccine confidence among New Jersey secondary and high school teachers based on survey data							
**Vaccination Status**	**Vaccinated (n=215)**	**%**	**Not vaccinated or IPNA (n=18)**	**%**	**Fisher's Exact Test**	**Total**	**%**
What was important for you to know to make you more confident in the COVID-19 vaccine? (Check all that apply)							
Agencies approving the vaccines are following strict rules	79	36.7%	2	11.1%	0.04*	81	34.8%
Health agencies and WHO recommend the vaccine and agree it is the same	74	34.4%	2	11.1%	0.06	76	32.6%
It is impossible to get COVID-19 or any other disease from the vaccine itself or its components	39	18.1%	1	5.6%	0.32	40	17.2%
My risk of getting sick with COVID-19 is bigger than the risk of side effects from the vaccine	111	51.6%	5	27.8%	0.08	116	49.8%
The fast production of the vaccine did not compromise its safety	66	30.7%	5	27.8%	1.00	71	30.5%
The vaccine cannot cause any immediate or long-term injury	73	34.0%	5	27.8%	0.8	78	33.5%
The vaccine works in protecting me from COVID-19	107	49.8%	1	5.6%	<0.001***	108	46.4%
The vaccine works in stopping the transmission from one person to another	73	34.0%	1	5.6%	0.02*	74	31.8%
I do not need any other information	22	10.2%	3	16.7%	0.42	25	10.7%
Other	8	3.7%	2	11.1%	0.17	10	4.3%
**Booster Status**	**Taken (n=167)**	**%**	**Not Taken (n=48)**	**%**	**Fisher's Exact Test**	**Total**	**%**
What was important for you to know to make you more confident in the COVID-19 vaccine? (Check all that apply)							
Agencies approving the vaccines are following strict rules	63	37.7%	16	33.3%	0.61	79	36.7%
Health agencies and WHO recommend the vaccine and agree it is the same	66	39.5%	8	16.7%	0.003**	74	34.4%
It is impossible to get COVID-19 or any other disease from the vaccine itself or its components	33	19.8%	6	12.5%	0.29	39	18.1%
My risk of getting sick with COVID-19 is bigger than the risk of side effects from the vaccine	94	56.3%	17	35.4%	0.01*	111	51.6%
The fast production of the vaccine did not compromise its safety	50	29.9%	16	33.3%	0.72	66	30.7%
The vaccine cannot cause any immediate or long-term injury	57	34.1%	16	33.3%	1.00	73	34.0%
The vaccine works in protecting me from COVID-19	90	53.9%	17	35.4%	0.03*	107	49.8%
The vaccine works in stopping the transmission from one person to another	64	38.3%	9	18.8%	0.02*	73	34.0%
I do not need any other information	17	10.2%	5	10.4%	1.00	22	10.2%
Other	6	3.6%	2	4.2%	1.00	8	3.7%
**COVID-19 Diagnosis**	**Positive (n=106)**	**%**	**Not Positive (n=124)**	**%**	**Fisher's Exact Test**	**Total**	**%**
What was important for you to know to make you more confident in the COVID-19 vaccine? (Check all that apply)							
Agencies approving the vaccines are following strict rules	40	37.7%	41	33.1%	0.49	81	35.2%
Health agencies and WHO recommend the vaccine and agree it is the same	34	32.1%	42	33.9%	0.78	76	33.0%
It is impossible to get COVID-19 or any other disease from the vaccine itself or its components	16	15.1%	24	19.4%	0.49	40	17.4%
My risk of getting sick with COVID-19 is bigger than the risk of side effects from the vaccine	49	46.2%	65	52.4%	0.36	114	49.6%
The fast production of the vaccine did not compromise its safety	35	33.0%	36	29.0%	0.57	71	30.9%
The vaccine cannot cause any immediate or long-term injury	37	34.9%	41	33.1%	0.78	78	33.9%
The vaccine works in protecting me from COVID-19	40	37.7%	68	54.8%	0.01**	108	47.0%
The vaccine works in stopping the transmission from one person to another	28	26.4%	46	37.1%	0.09	74	32.2%
I do not need any other information	12	11.3%	13	10.5%	0.84	25	10.9%
Other	4	3.8%	6	4.8%	0.76	10	4.3%

Gender	Female (n=147)	%	Male (n=72)	%	Fisher's Exact Test	Total	%
What was important for you to know to make you more confident in the COVID-19 vaccine? (Check all that apply)							
Agencies approving the vaccines are following strict rules	53	36.1%	27	37.5%	0.88	80	36.5%
Health agencies and WHO recommend the vaccine and agree it is the same	53	36.1%	21	29.2%	0.36	74	33.8%
It is impossible to get COVID-19 or any other disease from the vaccine itself or its components	27	18.4%	13	18.1%	1.00	40	18.3%
My risk of getting sick with COVID-19 is bigger than the risk of side effects from the vaccine	84	57.1%	30	41.7%	0.04*	114	52.1%
The fast production of the vaccine did not compromise its safety	47	32.0%	21	29.2%	0.76	68	31.1%
The vaccine cannot cause any immediate or long-term injury	51	34.7%	25	34.7%	1.00	76	34.7%
The vaccine works in protecting me from COVID-19	72	49.0%	34	47.2%	0.89	106	48.4%
The vaccine works in stopping the transmission from one person to another	51	34.7%	21	29.2%	0.45	72	32.9%
I do not need any other information	14	9.5%	8	11.1%	0.81	22	10.0%
Other	4	2.7%	3	4.2%	0.69	7	3.2%
IPNA	7	4.8%	3	4.2%	1.00	10	4.6%
**Race**	**Non-Hispanic White (n=174)**	**%**	**Other (n=39)**	**%**	**Fisher's Exact Test**	**Total**	**%**
What was important for you to know to make you more confident in the COVID-19 vaccine? (Check all that apply)							
Agencies approving the vaccines are following strict rules	65	37.4%	13	33.3%	0.72	78	36.6%
Health agencies and WHO recommend the vaccine and agree it is the same	59	33.9%	15	38.5%	0.58	74	34.7%
It is impossible to get COVID-19 or any other disease from the vaccine itself or its components	32	18.4%	8	20.5%	0.82	40	18.8%
My risk of getting sick with COVID-19 is bigger than the risk of side effects from the vaccine	95	54.6%	16	41.0%	0.16	111	52.1%
The fast production of the vaccine did not compromise its safety	53	30.5%	12	30.8%	1.00	65	30.5%
The vaccine cannot cause any immediate or long-term injury	62	35.6%	10	25.6%	0.27	72	33.8%
The vaccine works in protecting me from COVID-19	88	50.6%	15	38.4%	0.21	103	48.4%
The vaccine works in stopping the transmission from one person to another	62	35.6%	9	23.1%	0.19	71	33.3%
I do not need any other information	16	9.2%	8	20.5%	0.05*	24	11.3%
Other	5	2.9%	1	2.6%	1.00	6	2.8%
IPNA	7	4.0%	2	9.1%	0.67	9	4.2%
**Counties**	**Cumberland and Ocean (n=24)**	**%**	**Other Counties (n=205)**	**%**	**Fisher's Exact Test**	**9**	**%**
What was important for you to know to make you more confident in the COVID-19 vaccine? (Check all that apply)							
Agencies approving the vaccines are following strict rules	8	33.3%	73	35.6%	1.00	81	35.4%
Health agencies and WHO recommend the vaccine and agree it is the same	8	33.3%	68	33.2%	1.00	76	33.2%
It is impossible to get COVID-19 or any other disease from the vaccine itself or its components	7	29.2%	33	16.1%	0.15	40	17.5%
My risk of getting sick with COVID-19 is bigger than the risk of side effects from the vaccine	12	50.0%	104	50.7%	1.00	116	50.7%
The fast production of the vaccine did not compromise its safety	6	25.0%	63	30.7%	0.64	69	30.1%
The vaccine cannot cause any immediate or long-term injury	8	33.3%	68	33.2%	1.00	76	33.2%
The vaccine works in protecting me from COVID-19	14	58.3%	94	45.9%	0.28	108	47.2%
The vaccine works in stopping the transmission from one person to another	5	20.8%	69	33.7%	0.25	74	32.3%
I do not need any other information	4	16.7%	21	10.2%	0.31	25	10.9%
Other	1	4.2%	7	3.4%	0.59	8	3.5%

Note: IPNA is “I prefer not to answer”. *p=<0.05, ** p=<0.01, ***p=<0.001.
Note: Two people who did not get the vaccine chose “Agencies approving the vaccines are following strict rules” and “Health agencies and WHO recommend the vaccine and agree it is the same”.
Note: One person who did not get the vaccine chose “It is impossible to get COVID-19 or any other disease from the vaccine itself or its components”, “The vaccine works in protecting me from COVID-19” and “The vaccine works in stopping the transmission from one person to another”.
Note: This study was not able to delineate the number of booster shots gotten at the time of taking the survey.
Note: For all questions, zero to ten participants chose IPNA or did not answer.
Note: No positive diagnosis consists of both a negative diagnosis and “I do not know”. I do not know means either an inconclusive test or did not take a test.
Note: “Other” consisted of American Indian or Alaskan Native, Middle Eastern or North African, Hispanic Asian, Hispanic Black, Hispanic White, Non-Hispanic Asian, Non-Hispanic Black, those who indicated they were multiracial, and those who self-identified as Other.

Supplemental Table S4: Important factors regarding the COVID-19 vaccine among New Jersey secondary and high school teachers based on survey data

**Vaccination Status**	**Vaccinated (n=215)**	**%**	**Not vaccinated or IPNA (n=18)**	**%**	**Fisher's Exact Test**	**Total**	**%**
What else was important for you to know that made you more likely to take the COVID-19 vaccine? (Check all that apply)							
Everybody will have equal access to the vaccine regardless of income or race	74	34.4%	1	5.6%	0.01^{**}	75	32.2%
I will be free to choose if I get the vaccine or not with no consequences	40	18.6%	10	55.6%	0.001^{***}	50	21.5%
Once vaccinated I will be able to live my life with no restrictions	103	47.9%	1	5.6%	0.002^{**}	104	44.6%
Pharmaceutical companies will not make large profits from the vaccine	31	14.4%	2	11.1%	1.00	32	13.7%
The school district or school I work for required the vaccine	58	27.0%	1	5.6%	0.05^{*}	59	25.3%
There are no other reasons why so many people are sick	9	4.2%	1	5.6%	0.56	10	4.3%
Those with concerns about the vaccine have opportunities to share their opinions with the public	11	5.1%	2	11.1%	0.26	13	5.6%
**Booster Status**	**Taken (n=167)**	**%**	**Not Taken (n=48)**	**%**	**Fisher's Exact Test**	**Total**	**%**
What else was important for you to know that made you more likely to take the COVID-19 vaccine? (Check all that apply)							
Everybody will have equal access to the vaccine regardless of income or race	66	39.5%	8	16.7%	0.003^{**}	74	34.4%
I will be free to choose if I get the vaccine or not with no consequences	25	15.0%	15	31.3%	0.02^{*}	40	18.6%
Once vaccinated I will be able to live my life with no restrictions	84	50.3%	19	39.6%	0.25	103	47.9%
Pharmaceutical companies will not make large profits from the vaccine	22	13.2%	9	18.8%	0.35	31	14.4%
The school district or school I work for required the vaccine	39	23.4%	19	39.6%	0.04^{*}	58	27.0%
There are no other reasons why so many people are sick	8	4.8%	1	2.1%	0.69	9	4.2%
Those with concerns about the vaccine have opportunities to share their opinions with the public	9	5.4%	2	4.2%	1.00	11	5.1%
**Gender**	**Female (n=147)**	**%**	**Male (n=72)**	**%**	**Fisher's Exact Test**	**Total**	**%**
What else was important for you to know that made you more likely to take the COVID-19 vaccine? (Check all that apply)							
Everybody will have equal access to the vaccine regardless of income or race	51	34.7%	23	31.9%	0.76	74	33.8%
I will be free to choose if I get the vaccine or not with no consequences	31	21.1%	17	23.6%	0.73	48	21.9%
Once vaccinated I will be able to live my life with no restrictions	63	42.9%	39	54.2%	0.15	102	46.6%
Pharmaceutical companies will not make large profits from the vaccine	24	16.3%	9	12.5%	0.55	33	15.1%
The school district or school I work for required the vaccine	35	23.8%	21	29.2%	0.41	56	25.6%
There are no other reasons why so many people are sick	6	4.1%	4	5.6%	0.73	10	4.6%
Those with concerns about the vaccine have opportunities to share their opinions with the public	8	5.4%	5	6.9%	0.76	13	5.9%
**Race**	**Non-Hispanic White (n=174)**	**%**	**Other (n=39)**	**%**	**Fisher's Exact Test**	**Total**	**%**
What else was important for you to know that made you more likely to take the COVID-19 vaccine? (Check all that apply)							
Everybody will have equal access to the vaccine regardless of income or race	61	35.1%	12	30.8%	0.71	73	34.3%
I will be free to choose if I get the vaccine or not with no consequences	29	16.7%	16	41.0%	0.002^{**}	45	21.1%
Once vaccinated I will be able to live my life with no restrictions	84	48.3%	15	38.5%	0.29	99	46.5%
Pharmaceutical companies will not make large profits from the vaccine	27	15.5%	6	15.4%	1.00	33	15.5%
The school district or school I work for required the vaccine	46	26.4%	9	23.1%	0.84	55	25.8%
There are no other reasons why so many people are sick	8	4.6%	2	5.1%	1.00	10	4.7%
Those with concerns about the vaccine have opportunities to share their opinions with the public	9	5.2%	4	10.3%	0.26	13	6.1%

Counties	Cumberland and Ocean (n=24)	%	Other Counties (n=205)	%	Fisher's Exact Test	Total	%
What else was important for you to know that made you more likely to take the COVID-19 vaccine? (Check all that apply)							
Everybody will have equal access to the vaccine regardless of income or race	5	20.8%	70	34.1%	0.25	75	32.8%
I will be free to choose if I get the vaccine or not with no consequences	6	25.0%	44	21.5%	0.79	50	21.8%
Once vaccinated I will be able to live my life with no restrictions	16	66.7%	88	42.9%	0.03*	104	45.4%
Pharmaceutical companies will not make large profits from the vaccine	3	12.5%	30	14.6%	1	33	14.4%
The school district or school I work for required the vaccine	6	25.0%	52	25.4%	1	58	25.3%
There are no other reasons why so many people are sick	3	12.5%	7	3.4%	0.07	10	4.4%
Those with concerns about the vaccine have opportunities to share their opinions with the public	1	4.2%	12	5.9%	1	13	5.7%

Note: IPNA is "I prefer not to answer". *p=<0.05, ** p=<0.01, ***p=<0.001.
Note: No positive diagnosis consists of both a negative diagnosis and "I do not know". We can not determine if this means an inconclusive test or did not take a test.
Note: For all questions, eight to ten participants chose IPNA or did not answer.
Note: For people who have not been vaccinated or IPNA, only one participant chose the following options: "Once vaccinated I will be able to live my life with no restrictions", "Everybody will have equal access to the vaccine regardless of income or race", "There are no other reasons why so many people are sick", "The school district or school that I work for required the vaccine",
Note: Two chose "Those with concerns about the vaccine have opportunities to share their opinions with the public", and "Pharmaceutical companies will not make large profits from the vaccine".
Note: This study was not able to delineate the number of booster shots gotten at the time of taking the survey.
Note: One participant in the "No booster" category chose "There are no other reasons why so many people are sick" and two chose "Those with concerns about the vaccine have opportunities to share their opinions with the public". Note: No positive diagnosis consists of both a negative diagnosis and "I do not know". I do not know means either an inconclusive test or did not take a test.
Note: "Other" consisted of American Indian or Alaskan Native, Middle Eastern or North African, Hispanic Asian, Hispanic Black, Hispanic White, Non-Hispanic Asian, Non-Hispanic Black, those who indicated they were multiracial, and those who self-identified as Other.
Note: Two participants in the "Other" category chose "Those with concerns about the vaccine have opportunities to share their opinions with the public"
Note: Cumberland and Ocean counties are separated out because they have lower overall vaccination rates.
Note: One participant in the "Cumberland and Ocean" county chose "Those with concerns about the vaccine have opportunities to share their opinions with the public"

**Supplemental Table S5**: COVID-19 case information among New Jersey secondary and high school teachers based on survey data							
**Diagnosis Status**	**Positive (n=106)**	**%**	**Not Positive (n=124)**	**%**	**Fisher's Exact Test**	**Total**	**%**
Do you have any close family members older than 70 years?							
Yes, living together	14	13.2%	15	12.1%	0.72	29	12.6%
Yes, not living together	72	67.9%	80	64.5%		152	66.1%
No	20	18.9%	29	23.4%		49	21.3%
Do you personally know anyone in your family, group of friends, or community networks who became seriously ill or died as a result of COVID-19?							
Yes	64	60.4%	77	62.1%	0.89	141	61.3%
No	41	38.7%	47	37.9%		88	38.3%
Describe the level of care you received, or are receiving (n=104)							
Did not seek medical care	64	61.5%	0	0.0%	---	64	61.5%
Received medical care but was not hospitalized	37	35.6%	0	0.0%		37	35.6%
**Vaccination Status**	**Vaccinated (n=215)**	**%**	**Not vaccinated or IPNA (n=18)**	**%**	**Fisher's Exact Test**	**Total**	**%**
Do you have any close family members older than 70 years?							
Yes, living together	29	13.5%	0	0.0%	0.09	29	12.4%
Yes, not living together	138	64.2%	16	88.9%		154	66.1%
No	47	21.9%	2	11.1%		49	21.0%
Do you personally know anyone in your family, group of friends, or community networks who became seriously ill or died as a result of COVID-19?							
Yes	133	61.9%	8	44.4%	0.14	141	60.5%
No	80	37.2%	10	55.6%		90	38.6%
To your knowledge, do you have or have you had COVID-19?							
Yes	92	42.8%	14	77.8%	0.02*	106	45.5%
No	102	47.4%	3	16.7%		105	45.1%
I do not know	18	8.4%	1	5.6%		19	8.2%
Describe the level of care you received, or are receiving (n=106)							
Did not seek medical care	56	60.9%	8	57.1%	0.37	64	60.4%
Received medical care but was not hospitalized	34	37.0%	3	21.4%		37	34.9%
**Booster Status**	**Taken (n=167)**	**%**	**Not Taken (n=48)**	**%**	**Fisher's Exact Test**	**Total**	**%**
Do you have any close family members older than 70 years?							
Yes, living together	25	15.0%	4	8.3%	0.13	29	13.5%
Yes, not living together	101	60.5%	37	77.1%		138	64.2%
No	40	24.0%	7	14.6%		47	21.9%
Do you personally know anyone in your family, group of friends, or community networks who became seriously ill or died as a result of COVID-19?							
Yes	104	62.3%	29	60.4%	0.74	133	61.9%
No	61	36.5%	19	39.6%		80	37.2%
To your knowledge, do you have or have you had COVID-19?							
Yes	60	35.9%	32	66.7%	<0.001***	92	42.8%
No	88	52.7%	14	29.2%		102	47.4%
I do not know	16	9.6%	2	4.2%		18	8.4%
Describe the level of care you received, or are receiving (n=106)							
Did not seek medical care	37	61.7%	19	59.4%	0.92	56	60.9%
Received medical care but was not hospitalized	21	35.0%	13	40.6%		34	37.0%

Number of Vaccine Doses Received	One dose (n=14)	%	Two doses (n=201)	%	Fisher's Exact Test	Total	%
Do you have any close family members older than 70 years?							
Yes, living together	2	14.3%	27	13.4%	1.00	29	13.5%
Yes, not living together	9	64.3%	129	64.2%		138	64.2%
No	3	21.4%	44	21.9%		47	21.9%
Do you personally know anyone in your family, group of friends, or community networks who became seriously ill or died as a result of COVID-19?							
Yes	9	64.3%	124	61.7%	1.00	133	61.9%
No	5	35.7%	75	37.3%		80	37.2%
To your knowledge, do you have or have you had COVID-19?							
Yes	7	50.0%	85	42.3%	0.10	92	42.8%
No	4	28.6%	98	48.8%		102	47.4%
I do not know	3	21.4%	15	7.5%		18	8.4%
Describe the level of care you received, or are receiving (n=106)							
Did not seek medical care	6	85.7%	50	58.8%	0.21	56	60.9%
Received medical care but was not hospitalized	1	14.3%	33	38.8%		34	37.0%
**Gender**	**Female (n=147)**	**%**	**Male (n=72)**	**%**	**Fisher's Exact Test**	**Total**	**%**
Do you have any close family members older than 70 years?							
Yes, living together	19	12.9%	8	13.8%	0.95	27	12.3%
Yes, not living together	97	66.0%	49	84.5%		146	66.7%
No	30	20.4%	15	25.9%		45	20.5%
Do you personally know anyone in your family, group of friends, or community networks who became seriously ill or died as a result of COVID-19?							
Yes	90	61.2%	44	61.1%	1	134	61.2%
No	56	38.1%	27	37.5%		83	37.9%
To your knowledge, have you gotten COVID-19?							
Yes	68	46.3%	30	41.7%	0.59	98	44.7%
No	66	44.9%	35	48.6%		101	46.1%
I don't know	10	6.8%	7	9.7%		17	7.8%
Describe the level of care you received, or are receiving:							
Did not seek medical care	44	29.9%	16	22.2%	0.38	60	27.4%
Received medical care but was not hospitalized	21	14.3%	14	19.4%		35	16.0%
Was hospitalized	1	0.7%	0	0.0%		1	0.5%

Race	Non-Hispanic White (n=174)	%	Other (n=39)	%	Fisher's Exact Test	Total	%
Do you have any close family members older than 70 years?							
Yes, living together	20	11.4%	6	15.4%	0.37	26	12.2%
Yes, not living together	118	67.8%	22	56.4%		140	65.7%
No	35	20.1%	11	28.2%		46	21.5%
Do you personally know anyone in your family, group of friends, or community networks who became seriously ill or died as a result of COVID-19?							
Yes	100	57.4%	28	71.8%	0.15	128	60.1%
No	72	41.4%	11	28.2%		83	39.0%
To your knowledge, have you gotten COVID-19?							
Yes	77	44.2%	16	41.0%	0.93	93	43.7%
No	81	46.6%	20	51.2%		101	47.4%
I don't know	13	7.5%	3	7.7%		16	7.5%
Describe the level of care you received, or are receiving:							
Did not seek medical care	47	27.0%	8	20.5%	0.66	55	25.8%
Received medical care but was not hospitalized	27	15.5%	8	20.5%		35	16.4%
**County**	**Cumberland and Ocean (n=24)**	**%**	**Other Counties (n=205)**	**%**	**Fisher's Exact Test**	**Total**	**%**
Do you have any close family members older than 70 years?							
Yes, living together	5	20.8%	23	11.2%	0.37	28	12.2%
Yes, not living together	14	58.3%	138	67.3%		152	66.4%
No	5	20.8%	43	21.0%		48	21.0%
Do you personally know anyone in your family, group of friends, or community networks who became seriously ill or died as a result of COVID-19?							
Yes	13	54.2%	126	61.5%	0.51	139	60.7%
No	11	45.8%	77	37.6%		88	38.4%
To your knowledge, have you gotten COVID-19?							
Yes	14	58.3%	90	43.9%	0.48	104	45.4%
No	9	37.5%	94	45.9%		103	45.0%
I don't know	1	4.2%	18	8.8%		19	8.3%
Describe the level of care you received, or are receiving:							
Did not seek medical care	8	33.3%	56	31.8%	0.85	64	27.9%
Received medical care but was not hospitalized	6	25.0%	31	17.6%		37	16.2%

Note: IPNA is "I prefer not to answer". *p=<0.05, , ***p=<0.001.
Note: No positive diagnosis consists of both a negative diagnosis and "I do not know". We cannot determine if this means an inconclusive test or did not take a test.
Note: One participant belonging to the "Positive COVID diagnosis" group was hospitalized.
Note: One participant belonging to the "People who have been vaccinated" group was hospitalized.
Note: Two participants who have not been vaccinated reported they do not live with any family members older than 70 years.
Note: This study was not able to delineate the number of booster shots a participant had gotten at the time of taking the survey.
Note: One participant belonging to the "Booster taken" group was hospitalized.
Note: One participant belonging to the "Two doses" group was hospitalized.
Note: Two participants who received one dose of the COVID-19 vaccine reported they do live with family members older than 70 years
Note: "Other" consisted of American Indian or Alaskan Native, Middle Eastern or North African, Hispanic Asian, Hispanic Black, Hispanic White, Non-Hispanic Asian, Non-Hispanic Black, those who indicated they were multiracial, and those who self-identified as Other.
Note: One participant belonging to the "Female" group was hospitalized.
Note: One participant belonging to the "Non-Hispanic White" group was hospitalized.
Note: One participant belonging to the "Other Counties" group was hospitalized.
Note: One to 123 participants selected "I prefer not to answer" or did not answer this set of questions.
